# Effects of a 12-hour shift system on sleep and cardiovascular health of male machine and plant operators – a longitudinal study over four years

**DOI:** 10.3389/fpubh.2025.1616810

**Published:** 2025-08-28

**Authors:** Reingard Seibt, Steffi Kreuzfeld, Bettina Hunger

**Affiliations:** ^1^Institute for Occupational, Social and Environmental Medicine of the Rostock University Medical Centre, University of Rostock, Rostock, Germany; ^2^Office of ASD*BGN Coordination Berlin, German Social Accident Insurance Institution for the Foodstuffs and Catering Industry (BGN), Government Safety Organization Foods and Restaurants, Mannheim, Germany

**Keywords:** shift work, day work, longitudinal section, sleep, health, work-life balance

## Abstract

**Background:**

Data on the risks and effects of shift systems involving night work are inconsistent. In particular, there is a lack of longitudinal studies on the impact of 12-h shift systems on indicators of sleep, cardiovascular health and work-life balance. Therefore, this study compared machine and plant operators (MPO) who worked in a rotating 12-h shift system or only during the day, both at baseline (T1) and at follow-up 4 years later (T5).

**Methods:**

Data were collected annually and included a questionnaire on shift work and sleep as well as a cardiovascular screening programme. The sample for analysis consisted of 45 shift (SW) and 30 day workers (DW) (mean age T1: 40 years). Sleep behaviour was examined by sleep quality and quantity (PSQI score), cardiovascular health by blood pressure, body mass index (BMI), blood lipids, glycosylated haemoglobin (HbA1c) and PROCAM score. Work-life balance was assessed on the basis of life satisfaction and impairments. Analyses of covariance with repeated measures were used to determine longitudinal changes in the indicators between T1 and T5.

**Results:**

At T1, SW showed significantly poorer sleep quality (*d* = 0.58) and shorter sleep duration (*M* = 366 min vs. 438 min, *d* = 1.38) compared to DW. These effects increased significantly in SW only after night shifts at T5 (*M* = 5.1 pts, *η*^2^_p_ = 0.13, sleep duration: *M* = 318 min). At T1, SW differed from DW only by a significantly higher blood pressure (*d* = 0.60/0.49), BMI (*d* = 0.68) and PROCAM score in trend (*p* = 0.122). Lipids and HbA1c were comparable between the two groups. The means of the PROCAM score were in the low-moderate range, predicting a risk of heart attack <10% for 87% of the MPOs. At T5, the group differences for cardiovascular health from T1 were confirmed. SW achieved significantly higher satisfaction at T5 (*η*^2^_p_ = 0.22); it corresponded to that of DW. Both groups reported significantly fewer impairments at T5 (*d* = 0.68/0.58).

**Conclusion:**

At T5, the 12-h shift system demonstrably changed sleep behaviour but not cardiovascular health. Sleep deficits could not be compensated. The 12-h shift system seems to offer advantages for work-life balance.

## 1 Introduction

The current labour market continues to be characterised by traditional demands on shift systems with night and weekend work. In certain sectors of the economy, shift work is essential to maintain production processes. In 2020, 13% of men in Germany worked night shifts and 15% worked rotating shifts (1).

Shift and night work are defined differently internationally, and studies often use the terms imprecisely (2, 3). The different use of the term in the primary studies of the meta-analysis by Ijaz et al. (4) prevents a comprehensive assessment of the effects of shift and night work on sleep, health or work-life balance. In Germany, night work is regulated by § 2 of the Working Hours Act (ArbZG) (5). It covers the period from 23:00 h to 06:00 h and applies to any work that extends more than 2 h into the night. In addition, in Germany the eight-hour day also applies to shift workers. The German Working Time Act (§ 7, § 15, para. 1) only allows a continuous 12-h shift system under certain conditions and with additional regulations.

Although there are concerns about such shift systems for reasons of productivity and performance, there is an interest in 12-h shifts from the perspective of both companies and shift workers themselves (6). Employers expect benefits for operational processes and employees expect more free time (7, 8). Overall, the data on the consequences of shift and night work is extensive but inconsistent, which is not least due to the lack of a standardised definition of shift and night work (3, 9) and the large number of different shift systems (10).

Obtaining enough high-quality sleep is essential for good health. However, night work leads to a physiological desynchronization of bodily functions (2, 9, 11–13). The change in sleep–wake rhythm not only causes additional burden but is also associated with a variety of impairments, most commonly sleep deficits and sleep disorders (8, 12–17). This leads to sleep-related mood disorders and a lack of recovery (e.g., daytime sleepiness, irritability, concentration problems) (14, 18), which increase the risk of errors and work-related accidents (14, 16, 18). It has been observed particularly in occupations with a high mental workload after more than 8 h of work (6, 18).

Numerous studies have associated night and rotating shift work with an increased risk of cardiovascular disease (9, 16, 19, 20) and metabolic diseases (9, 16, 20–22). The elevated risk of cardiovascular disease (15, 23) and type II diabetes (9, 15, 22, 23) has also been derived from meta-analyses and multi-cohort studies for long working hours. However, other reviews and studies have not provided convincing evidence of a link between shift work and cardiovascular disease (24–26) or type II diabetes (21). The longitudinal study (five-year follow-up) by Rossnagel et al. (27) also found no significant association between long working hours and cardiovascular disease or type II diabetes.

The contradictory data also applies to cardiovascular risk factors. While a number of studies (9, 11, 28–31) and the systematic review by Esquirol et al. (2) found an association between shift and night work and hypertension, longitudinal studies by Morikawa et al. (32) or Akbari et al. (28) or the cohort study by Hublin et al. (24) did not confirm this correlation.

Night work in particular has been associated with an increased risk of being overweight and obesity in several systematic reviews (33–35). In contrast, no such association was identified in the cohort study by Akbari et al. (28) or the meta-analysis by Saulle et al. (36). Other studies and the review by Esquirol et al. (2) have shown that shift and night workers have raised lipid levels (total cholesterol, low-density lipoprotein and high-density lipoprotein cholesterol, triglycerides) (31, 37) and glucose levels (fast blood sugar, fasting plasma glucose) (38), while in the studies by Morikawa et al. (32), Akbari et al. (28) or Dong et al. (30), indicators of lipid and glucose metabolism did not differ between shift and day workers.

It has also been observed that long working hours, shift and night work lead to changes in lifestyle and health behaviour (9, 12, 39). For example, it has been found that shift workers have unfavourable eating habits (22, 25), smoke more often (22, 25, 35) and are less physically active (22, 36, 40) compared to day workers. Other studies have found no evidence between shift work and physical inactivity (41, 42) or smoking (32).

Another well-known consequence of shift and night work is social desynchronization, which manifests in restrictions on family and social life (7, 25, 43, 44) and less participation in social activities. Night and weekend shifts, in particular, are at odds with the desire for evenings, weekends and holidays off (7). However, the results on the impact on work-life balance have also been found to be contradictory (7), with the duration and organisation of working time, as well as the opportunities for employee participation, moderating the result (10). Although long working hours appeared to make it more difficult to reconcile work and private life (45), a better work-life balance was confirmed for the 12-h shift system (46). Loudoun (47), on the other hand, found no difference in work-life balance for an eight-hour or 12-h shift system.

To summarise, there is a lack of meaningful longitudinal studies that address the influence of 12-h shift systems on indicators of sleep, cardiovascular health and work-life balance. In addition, the findings are inconsistent due to the heterogeneity of the methodology (e.g., definition of shift work, design, population, data collection, adjustment for potential covariates). Furthermore, the occupational group of machine and plant operators (MPO) is clearly underrepresented in the literature.

MPOs are primarily found in industrial production sectors (including the food industry). They are involved in the entire production process and must guarantee smooth production. Their range of tasks includes setting up, commissioning, operating and maintaining machines and production facilities, selecting and applying mechanical and manual production techniques, controlling the flow of materials and performing quality control and assurance tasks (48). In addition, there is coordination with upstream and downstream production levels and the documentation of production data.

For technological and economic reasons, MPOs requires a high proportion of night shift work, although two-shift operation is more common on weekdays in the food and beverage industry. Although a 12-h shift system increases the degree of utilisation of plant capacities, higher labour costs are incurred.

One of the primary stress factors for MPOs is the intense, sustained and concentrated attention required to capture, process and implement relevant process information. This can lead to changes in performance over the course of the day, with a particular drop in performance at night (49). Since the operating and control processes are mainly carried out while standing, a solid physical fitness is required. Good stress management is also essential, as maintenance and repair work often has to be carried out under time pressure (50).

The aim of the study was therefore to map the development of indicators of sleep, cardiovascular health and work-life balance after a period of 4 years (follow-up measurement T5) in MPOs working in a rotating 65-h shift system or exclusively during the day (8 h) in comparison to the baseline measurement (T1). It was hypothetically assumed that rotating 12-h shift work over a period of 4 years would have a negative impact on sleep, cardiovascular health and work-life balance under comparable baseline conditions for the two groups.

## 2 Methods

This study was a longitudinal study with a baseline measurement (T1) and follow-up after 4 years (T5). The data was collected annually from 2011 (first measurement point, T1) to 2016 (fifth measurement point, T5) at a company in the food industry in Saxony-Anhalt (>400 examinations). The 12-h shift system was introduced at this company in November 2009. Interim results (T2-T4) are not reported here since they do not provide any additional insights.

A total of 108 employees took part in the study, of whom 75 datasets from male MPOs were available which met the data quality requirements, i.e., 45 shift workers and 30 day workers (working regular daytime hours) took part in both the baseline and the follow-up measurement after 4 years; they form the data basis of this study. Since there were only four female MPOs in the baseline measurement, no women were included in the sample.

The examinations were carried out by THUMEDI Präventionsmanagement GmbH as part of the occupational health programme for shift workers. The examinations were carried out in the company’s medical centres, with each participant being examined for 30 min. All participants took part in the examinations voluntarily. Before the start of the examination, all employees received a letter with information on data protection, the conduct of the examination and data analysis. The anonymity of the data was guaranteed by means of a personal code.

### 2.1 Research methods

The screening concept was developed by THUMEDI Präventionsmanagement GmbH and the Psychophysiological Diagnostics Department of the Faculty of Medicine at the Technical University of Dresden (51) and consisted of a modified shift work questionnaire (52), other standardised survey instruments, additional questions and a cardiovascular screening programme. The shift work questionnaire was completed in advance of the examination and brought to the occupational health screening programme. This made it possible to check the completeness of the data on site; inaccuracies could be corrected and missing information completed. Internal consistencies of the scales in the questionnaires were analysed using Cronbach’s alpha and evaluated according to Blanz (53).

#### 2.1.1 Shift work questionnaire

The Shift Work Questionnaire (SWQ) was developed based on the international standard Shiftwork Index [SSI, (54)] and reduced to reflect the specific characteristics of the MPO job profile (52). The SSI includes the following six sections, each focusing on a specific aspect of shift work: 1. general biographical information, 2. sleep and fatigue, 3. health and well-being, 4. social and domestic situation, 5. coping strategies, 6. chronotype and personality. A comprehensive description can be found in Barton et al. (54). In this study, selected questions from the SSI were used to examine the family situation [e.g., gender, age, marital status, children in the household (yes/no, number)] and shift-related workload (e.g., job title, years in shift work, weekly working hours, shift system, shift duration) (section 1) as well as health factors [e.g., complaints, illnesses or diagnoses, medication) (section 3) and aspects of work-life balance (section 4)].

The questions on health behaviour (e.g., smoking status, alcohol consumption, sporting activity) were developed in-house. For smoking, respondents were asked whether they are or were smokers (*yes/no*) and the average number of cigarettes consumed per day was recorded. Former smokers were categorised as non-smokers. Questions on alcohol consumption related to the frequency (*not at all, occasionally, regularly*) and amount of alcohol consumed (converted into g/day). Sporting activity was determined on the basis of frequency (*not at all, occasionally, regularly/week*), the amount of time/week and the type of sport practised.

The work-life balance was determined on the basis of two scales: satisfaction with available time (satisfaction scale) and impairment of work and private life (impairment scale). The questions were based on aspects known from the literature on the social consequences of shift work. A total of 13 items were asked, with 8 items on the satisfaction scale and 5 items on the impairment scale. The items for the satisfaction scale were assessed using a three-point response scale (*1 = yes, 2 = partly, 3 = no*), while the items on impairment (work–family conflict or family–work conflict) were assessed on a five-point response scale (*1 = always, 2 = frequently, 3 = occasionally, 4 = rarely, 5 = never*), with scales 1 and 2 (*frequently*) and 4 and 5 (*never*) being summarised for the presentation of results. The satisfaction score (range: 8–24 pts) and the impairment score (range: 5–25 pts) were calculated by adding the respective item details. On the satisfaction scale, a low score indicated a high level of satisfaction with the free time available; on the impairment scale, a low score indicated frequent impairment of work and private life. The Cronbach’s alpha for the satisfaction and impairment scales was 0.92 and 0.73 respectively, which according to Blanz (53) corresponds to excellent and acceptable quality.

#### 2.1.2 Pittsburgh sleep quality index

The Pittsburgh Sleep Quality Index [PSQI, (55)] was used to measure sleep quality and quantity in the German translation by Riemann and Backhaus (56), adapted for shift workers (52). It consisted of 19 items, which were summarised into the following seven components: sleep quality, usual sleep times, latency to fall asleep and duration of sleep (sleep quantity), sleep disturbances, use of sleep medication, daytime sleepiness. Each component covered the value range from zero to three pts and was surveyed retrospectively for the last 4 weeks. The PSQI score results from the summation of the seven component scores and can vary between zero and 21 pts, with a high score corresponding to reduced sleep quality. A cut-off value of five pts allows differentiation between good and poor sleepers, and a cut-off value of 11 pts or more is assumed to indicate chronic sleep disorders (55). For shift workers, the items on sleeping and waking times as well as sleep duration in the morning and night shifts were differentiated and the PSQI score was determined for both shifts.

The PSQI has a high or acceptable reliability (57): Cronbach’s *α* were 0.85 and 0.75. For the present data, Cronbach’s alpha was 0.73 and is therefore an acceptable indicator of internal consistency (53).

#### 2.1.3 Morningness-Eveningness Questionnaire

To determine the individual chronotype, the validated German version of the Morningness-Eveningness Questionnaire [DMEQ, (58)] was used, which was originally developed as an English version by Horne and Ostberg (59).

The D-MEQ consists of 19 items with different Likert scales and records temporal preferences and habits of the sleep–wake cycle (e.g., preferred bed and getting-up/rising times), performance and subjective well-being. The answers were each given a point value and added up to a total score ranging from 16 to 86 points. Based on this, the following chronotypes were differentiated (58): definite morning type (70–86 pts), moderate morning type (59–69 pts), neutral type (42–58 pts), moderate evening type (31–41 pts), definite evening type (16–30 pts). Thus, a higher score indicates a preference for an earlier arising time, an earlier time for high performance, and an earlier finish time.

The retest reliability of the D-MEQ was reported as rtt = 0.96 (58), which can be classified as excellent or reliable (53). The internal consistency of the D-MEQ varies between 0.84 and 0.87 for Cronbach’s alpha (60), which is considered high (53). In the present study, a Cronbach’s alpha of 0.82 was determined for the D-MEQ, which is in the good range (53).

#### 2.1.4 Cardiovascular screening programme

The following examinations were carried out to assess cardiovascular health:

- blood pressure (BP) measurement
- physical examination (including determination of the body mass index)
- blood sampling (determination of indicators of lipid and glucose metabolism)
- calculation of the Prospective Cardiovascular Münster Score (PROCAM score).

##### 2.1.4.1 Blood pressure measurement

BP measurement was used in particular for the early detection of high BP (hypertension). The recommendations of Pickering et al. (61) were followed and a calibrated BP measuring device (BOSO medicus from Bosch + Sohn GmbH u. Co. KG) was used for the measurement (practice BP measurement). BP was measured by healthcare professionals after about 5 min of rest in a sitting position – on both the left and right upper arm; it was given in millimetres of mercury [mmHg]. After 2 min, a repeat measurement was taken on the arm with the higher BP, which was used to categorise normotensive and hypertensive MPOs. Hypertensive BP was defined as values ≥140/90 mmHg (or/and), normotensive BP was defined as values <140/90 mmHg (62). Even if hypertensive BP values measured once or twice are only suspected to be high BP, those affected were labelled as hypertensive in this study. Anyone taking antihypertensive medication was categorised as hypertensive per se.

##### 2.1.4.2 Physical examination

Body weight and height were measured according to the standardised STEPS protocol (63) and the body mass index (BMI) was calculated using the following formula: BMI = body weight [kg]/height squared [m^2^]. Based on the criteria of the German Obesity Society (64), the BMI was used to classify underweight (<18.5 kg/m^2^), normal weight (18.5–24.9 kg/m^2^), overweight (25.0–29.9 kg/m^2^) and obesity (≥30.0 kg/m^2^).

##### 2.1.4.3 Blood sampling

Total cholesterol (TC), low-density lipoprotein cholesterol (LDL-C) and high-density lipoprotein cholesterol (HDL-C), triglycerides (TG) were examined as indicators of lipid metabolism and fasting serum glucose and glycated haemoglobin A1c (HbA1c) as indicators of carbohydrate metabolism, whereby LDL-C, HDL-C and TG were used as components of the PROCAM score. For their determination, venous blood was taken from the crook of the arm. Blood collection and transport of the blood samples and their analyses in the laboratory were carried out according to defined standards. Blood samples were taken on the day of the study between 05:30 and 08:00 a.m. in a fasting state. The lipids were determined from the serum sample, the glycated haemoglobin from the plasma (anticoagulant ethylene diamine tetraacetic acid (EDTA)).

In practice, different standard ranges or cut-off values are known for the clinical interpretation of lipid and carbohydrate metabolism, whereby the diagnostic criteria vary depending on other risk factors and treatment goals. In addition, different units of measurement are used in the literature, which makes it difficult to compare the data. For example, the indicators of lipid metabolism are given in millimoles/litre [mmol/l] or milligrams/decilitre [mg/dl], HbA1c as a percentage of total haemoglobin [%] or in relation to 1 mole of haemoglobin in millimoles/mole of Hb [mmol/mol]. Irrespective of this, the classification of the American Association of Clinical Endocrinologists (AACE) (65) was used for the clinical interpretation of lipids (Table 1).

**Table 1:** Clinical evaluation of lipid metabolism indicators for men according to Jellinger et al. (65).

Category	Classification of the indicators of lipid metabolism (mg/dl [mmol/l])			
	Normal	Borderline	Elevated/decreased	High
Total cholesterol	<200 [<5.2]	200–239 [5.2–6.2]	>239 [>6.2]	
LDL cholesterol	<130 [<3.3]	130–159 [3.3–4.1]	>159–189 [>4.1–4.9]	>189 [>4.9]
HDL cholesterol	>60 [>1.5]	60–40 [1.5–1.0]	<40 [<1.0]	
Trigylcerides	<150 [<1.7]	150–199 [1.7–2.2]	>199–481 [>2.2–5.6]	>481 [>5.6]

The HbA1c value is the proportion of native haemoglobin with glucose binding and was examined to assess the risk or the presence of diabetes mellitus. It provides information on the level and duration of elevated glucose levels in the plasma over the last four to 12 weeks (long-term blood glucose value). In healthy adults, HbA1c values below 39 mmol/mol (<5.7%) are considered normal, HbA1c values between 39 and 48 mmol/mol (5.7–6.4%) indicate impaired blood glucose regulation (prediabetes), while HbA1c values above 48 mmol/mol (>6.4%) are assumed to indicate diabetes mellitus (64).

##### 2.1.4.4 PROCAM score

The PROCAM score (66) was used to estimate the ten-year risk of myocardial infarction. It is based on the epidemiological PROCAM study and applies to women and men aged between 20 and 75. The following eight factors are included in its calculation, each of which contributes independently to the individual risk of heart attack: age [years], gender, smoking status, presence of diabetes and/or heart attack in the family (first-degree relatives), systolic BP [mmHg] (taking into account the use of antihypertensives), blood concentration of HDL-C and LDL-C [mg/dl] and the TG [mg/dl]. Depending on the severity of the risk factors, different point values are assigned and then added to the PROCAM score (total score). According to Assmann et al. (66), 0–47 pts represent a low (<10%), 48–56 pts a medium (10–20%) and more than 56 pts a high PROCAM risk (>20%), i.e., the lower the PROCAM score, the lower the risk of suffering a heart attack in the next 10 years.

### 2.2 Data control and statistical analyses

Prior to the statistical calculations, the entire dataset was analysed for implausible information and statistical outliers in the data. There were no missing values for the questionnaire scales. For physiological measurements, missing values were replaced by the group mean. The statistical analyses of the data were carried out using the “Statistical Package for the Social Science” programme (SPSS, version 28) for Windows. First, the indicators for sleep, cardiovascular health and work-life balance were analysed descriptively (means, standard deviations, medians, quartiles, frequencies, percentages) and examined for differences between the two groups. Simple mean differences were analysed for continuous variables using the *t*-test for independent samples and for categorical variables using the *chi*^2^ test and the *contingency coefficient* (*CC*) (67). In addition, significance tests were carried out for continuous variables within a group for the survey time points (T1:T5) using the *t*-test for dependent samples.

Previous research has shown that the effects of shift work on sleep, health and work-life balance are affected by numerous confounding factors (covariates): *years in shift work, age, regular exercise, children in the household, chronotype*. In a preliminary analysis, associations between these covariates and the independent variables sleep, health and work-life balance, as well as between baseline and follow-up measurements, were analysed using Spearman’s rank correlation coefficient (*R*) and interpreted according to Bühl (68).

To analyse changes the indicators between the survey time points (T1:T5) in both groups, *two-factor analyses of covariance with repeated measures (rANCOVA*: within-subject factor *time,* between-subject factor *group*) were calculated. As a result of this correlation analysis, the covariates to be considered later in the *rANCOVA* were determined, with associations of *R* > 0.20 being considered relevant for inclusion as covariates.

The *analysis of variance* is considered to be relatively robust against violations of its application assumptions (normal distribution of dependent variables and variance homogeneity between the groups, *Levene* test) (67).

An error probability of *α* < 0.05 was defined as the statistical significance criterion and supplemented by effect sizes. Their interpretation was based on the conventions of Cohen (69). Small effect sizes from *d* ≥ 0.20 in the t-test or *χ*^2^-test and from *η*^2^_p_ ≥ 0.01 in the *rANCOVA* are considered practically relevant effects. The effect size *d* was calculated according to the formulas of Lenhard and Lenhard (70).

#### 2.2.1 Sample estimation

Before the start of the study, a sample estimation was carried out using the G*Power programme (71). It was expected that working in a rotating 12-h shift system over a period of 4 years would have a significant impact on sleep quality and quantity. However, we found no effect sizes in the few longitudinal studies that investigated the effects of shift work on sleep. We therefore used the longitudinal study by Åkerstedt et al. (72) as a reference, which found a significant increase in the risk of difficulty falling asleep [odds ratio (OR = 2.8, CI = 1.84.5) for shift work compared to day work.

Accordingly, an ANOVA with repeated measures over a period of 4 years (T1, T5) with an effect size of *η*^2^_p_ = 0.14 (corresponds to *f* = 0.403) and a power of 0.90 would require a total of 67 study participants to obtain a significant result (*α* = 0.05). A drop-out rate of 20% was assumed here. We have based this on the dropout rate from clinical studies and our own experience in shift work research.

## 3 Results

The study investigated whether work in a rotating 12-h shift system compared to day work over a period of 4 years (T5) led to poorer sleep quality and quantity, whether the cardiovascular risk indicators increased, or whether the work-life balance changed. Firstly, the sample is described. After that, the results of the baseline measurement (T1) are shown for each of these constructs in a comparison of shift and day workers (*t*-test for independent samples, *chi*^2^ test, *contingency coefficient*). Finally, the results of the repeat measurements (rANCOVAs) are presented for the respective constructs using the multivariate tests (Hotelling’s Trace).

The main and interaction effects between baseline and follow-up measurement (factor *time*) and the two groups (factor *group*), as well as the covariates are considered in the following.

As a result of the preliminary analysis, the covariates were included in the *rANCOVAs* as follows: *age* was included as a confounding variable for the sleep and cardiovascular health indicators (exception: for the PROCAM score). For the work-life balance the covariate children living in the household *(children/household [number]*) was included in the repeated measurement models. Since very high correlations (*R* ≥ 0.95) were confirmed for these covariates between T1 and T5, only the data from T1 were included in the analyses. The covariates *chronotype*, *years in shift work* (shift workers only) and *regular sport/week* were omitted as no practically significant correlations were found for these variables in the preliminary analysis for the sleep and health indicators.

### 3.1 Sample

The sample consisted of 45 MPOs who worked in a 12-h rotating shift system (06:00–18:00 h—morning shift, 18:00–06:00 h—night shift) with a night shift component and 30 MPOs who only worked 8 h during the day (06:00–14:30 h—day work), (hereinafter referred to as shift worker—SW vs. day worker—DW = groups). The 12-h shift system was a forward-rotating shift system with 1 day off after the morning shifts and 3 days off after the night shifts. All employees were employed full-time (40 h/week). In addition to MPOs, the group of day workers also included electrical fitters, maintenance mechanics and engineers (hereinafter referred to as MPO).

At the baseline measurement, the average age of the sample was 40 years. At this point, the shift workers had already been working shifts for an average of 9 years, while the day workers had been in their current job for around 1 year. They had always worked during the day in their previous careers. Overall, the MPOs had worked for an average of 23 years (*p* = 0.595).

#### 3.1.1 Relationship status

The two groups did not differ in marital status or children living in the household (*p* > 0.05). In the sample, 80% of the MPOs lived with a partner (T5: 83%); more than half of them (57%) had no children in the household (T5: 53%). On average, shift workers had two children living in their households, while day workers had just one (T5—SW vs. DW: 2 children vs. 1 child). Further characteristics of the sample can be found in Table 2.

**Table 2:** Sample characteristics of the shift and day workers at baseline.

Sample characteristic	Dimension	Shift work (*n* = 45)	Day work (*n* = 30)	Test-value	*p*-value	Effect size *d*
Personal and work-related status						
Age [years]	*M* ± SD	40.0 ± 10.4	40.6 ± 11.3	0.25	0.730	0.06
Duration of employment [years]	*M* ± SD	22.9 ± 10.8	21.4 ± 12.3	0.53	0.595	0.13
Duration of shift work [years]	*M* ± SD	8.7 ± 10.0				
	Mdn (Q_25_, Q_75_)	9 (5, 11)				
Relationship status						
Permanent partnership	*n* (%)	35 (77.8)	25 (83.3)	0.35	0.841	0.14
With parents/parents-in-law	*n* (%)	4 (8.9)	2 (6.7)			
Single	*n* (%)	6 (13.3)	3 (10.0)			
Children and relatives						
Children in the household [yes]	*n* (%)	22 (48.9)	10 (33.3)	1.78	0.182	0.31
Children in the household [number]	*M* ± SD	1.8 ± 0.8	1.0 ± 0.0	4.47	<0.001***	1.14
Care of relatives [yes]	*n* (%)	4 (9.8)	– (—)	0.28	0.093	0.12
Health behaviour						
Smoking status						
Smoking [yes]	*n* (%)	15 (33.3)	9 (30.0)	0.09	0.762	0.07
Cigarettes [number/day]	*M* ± SD	12.5 ± 6.8	11.2 ± 6.8	0.46	0.653	0.19
Alcohol use						
Regular alcohol consumption [yes]	*n* (%)	28 (62.2)	21 (70.0)	0.51	0.777	0.17
Quantity of alcohol [g/day]	*M* ± SD	11.0 ± 6.9	13.0 ± 6.7	1.02	0.157	0.29
Sporting activity						
Regular sport/week	*n* (%)	25 (56.6)	24 (80.0)	4.75	0.029*	0.52
Time for sport [min/week]	*M* ± SD	204.0 ± 121.9	238.8 ± 125.3	0.98	0.165	0.28
Chronotype						
Chronotype	*M* ± SD	58.6 ± 8.9	58.7 ± 8.7	0.07	0.945	0.02
Definitely evening type [16–30 pts]	*n* (%)	1 (2.2)	– (—)	4.81	0.308	0.52
Moderately evening type [31–41 pts]	*n* (%)	– (—)	(6.7)			
Neither type [42–58 pts]	*n* (%)	18 (40.0)	(46.7)			
Moderately morning type [59–69 pts]	*n* (%)	22 (48.9)	(43.3)			
Definitely morning type [70–86 pts]	*n* (%)	4 (8.9)	1 (3.3)			

*M* ± SD: mean ± standard deviation; Mdn (Q_25_, Q_75_): median (quartiles); *n* (%): sample size (frequency in percent). *t*-test (test size: *t*-value, effect size: *d*). *n* (%): *chi*^2^ test (test size: *χ*^2^-value, effect size: *d*). *p*-value: significance (two-sided): *p < 0.05, **p < 0.01. Effect size *d* according to Cohen (69): <0.20 = no effect, 0.20–0.49 = small effect, 0.50–0.79 = medium effect, ≥0.80 = large effect.

#### 3.1.2 Health behaviour

There were also no statistically significant differences for smoking and alcohol consumption (*p* > 0.05): 32% of the MPOs were smokers (T5: 31%) who smoked an average of 12 cigarettes/day (T5: M = 13 cigarettes/day). Overall, almost two thirds (65%) of the sample stated that they drank alcohol *regularly* (T5: 65%); 15% consumed alcohol *occasionally* (e.g., at parties). With regard to sporting activity, there was a significant difference (*d* = 0.52—medium effect) between the two groups: 57% of shift workers (T5: 60%) and 80°% of day workers (T5: 83%, *d* = 0.51—medium effect) exercised *regularly*, whereby the amount of time spent exercising did not differ between the two groups (*p* = 0.165) and averaged 204 and 239 min/week, respectively (T5: 217 and 207 min/week).

#### 3.1.3 Chronotype

The expression of the individual chronotype was comparable in both groups (*d* = 0.016). Their mean values ranged between ‘neutral chronotype’ and ‘moderate morning type’, with most shift workers (49%) classified as ‘moderate morning type’ and most day workers (47%) as ‘neutral chronotype’ (*p* = 0.308).

### 3.2 Sleep behaviour

**Figure 1:**
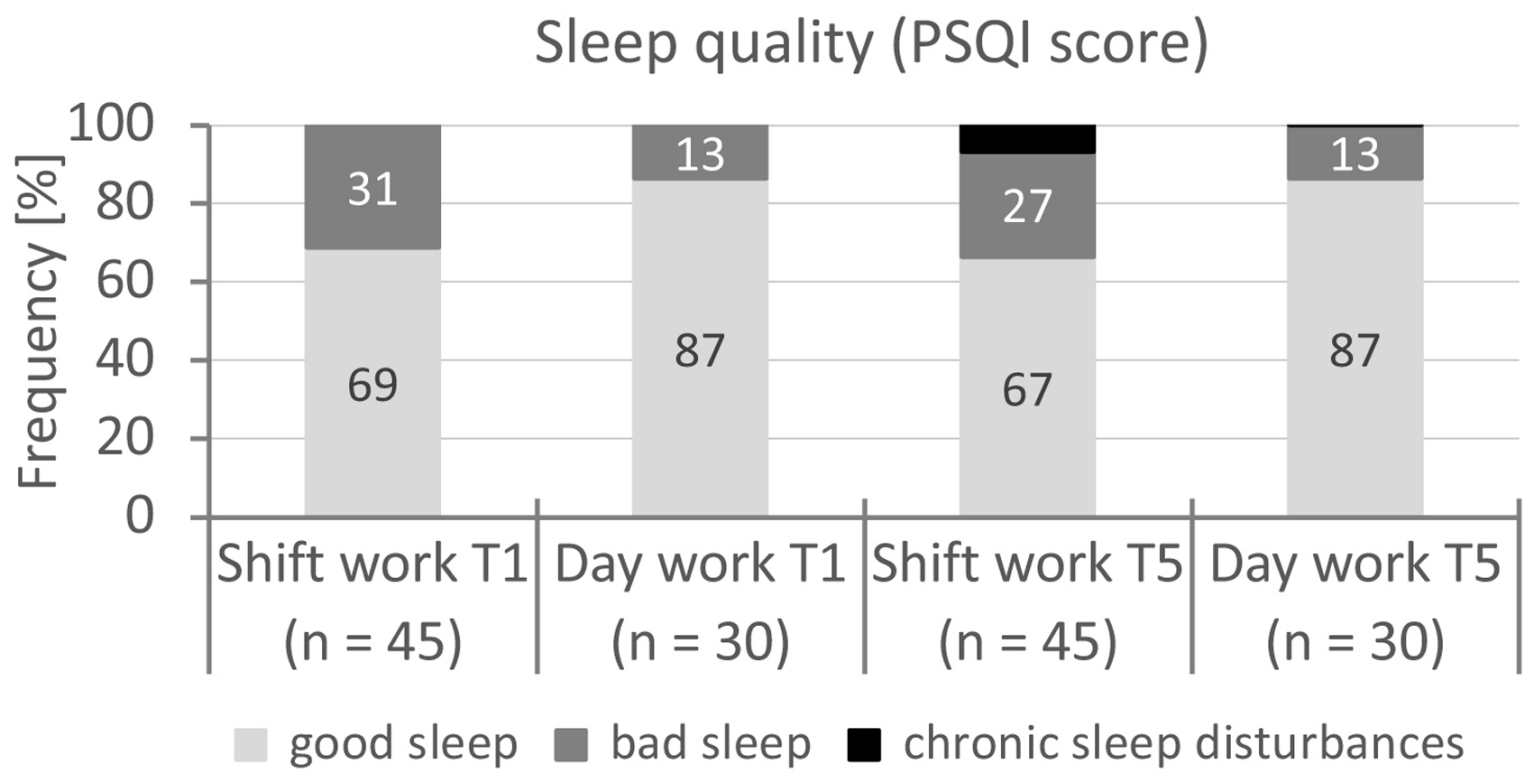
Classification of sleep quality (PSQI score) of shift and day workers at baseline and follow-up (T1, T5). Notes. PSQI: Pittsburgh Sleep Quality Index [PSQI score (pts)]. *n*: sample size. *chi*^2^ test (test size: *χ*^2^-value, effect size: Cohen’s *d*). *p*-value: significance (two-sided): *p* > 0.05 – no significance. Effect size according to Cohen (69): *d*: <0.20 = no effect, 0.20–0.49 = small effect, 0.50–0.79 = medium effect, ≥0.80 = large effect. Baseline: *χ*^2^(1) = 3.12, *p* = 0.077, *d* = 0.42, follow-up: *χ*^2^(1) = 4.46, *p* = 0.101, *d* = 0.50.

The results of the sleep behaviour for the baseline and follow-up are summarised in Table 3 and Figure 1.

**Table 3:** Main and interaction effects of sleep quality (PSQI score) of shift and day workers at baseline and follow-up.

Measurement time	Shift work (*n* = 45)	Day work (*n* = 30)	Within- and between-subject effects		
			Test-value	*p*-value	Effect size
Baseline (*M* ± SD) [pts]	4.4 ± 1.5	3.5 ± 1.7	*t* = 2.54	0.017*	*d* = 0.58
Follow-up (*M* ± SD) [pts]	4.8 ± 2.8	3.1 ± 2.0	*t* = 2.98	0.004**	*d* = 0.66
	Group		*F* = 9.05	0.004**	*η*^2^_p_ = 0.11
	Time		*F* = 2.72	0.137	*η*^2^*_p_* < 0.01
	Time * group		*F* = 2.06	0.155	*η*^2^_p_ = 0.03
Covariate	Age		*F* = 1.45	0.232	*η*^2^_p_ = 0.02

	Shift work				
	Morning shift (*n* = 45)	Night shift (*n* = 45)			
Baseline (*M* ± SD) [pts]	4.4 ± 1.5	4.0 ± 1.8	*t* = 1.73	0.091	*d* = 0.26
Follow-up (*M* ± SD) [pts]	4.8 ± 2.8	5.1 ± 2.4	*t* = 1.00	0.161	*d* = 0.15
	Shift		*F* = 0.47	0.496	*η*^2^_p_ = 0.01
	Time		*F* = 1.06	0.309	*η*^2^_p_ = 0.03
	Shift * time		*F* = 6.52	0.014*	*η*^2^_p_ = 0.13
Covariate	Age		*F* = 0.74	0.395	*η*^2^_p_ = 0.02

PSQ1: Pittsburgh Sleep Quality Index (PSQI score [pts]). *M* ± SD: mean ± standard deviation, *n*: sample size. *t*-test (test size: *t*-value, effect size: *d*). *rANCOVA* (test size: *F*-value, effect size: *η*^2^_p_): design: constant term + group + age; within-subjects effect: time (T1, T5). *p*-value: significance (two-sided): *p < 0.05, **p < 0.01. Effect size according to Cohen (69): *d*: <0.20 = no effect, 0.20–0.49 = small effect, 0.50–0.79 = medium effect, ≥0.80 = large effect. *η*^2^_p_: <0.01 = no effect, 0.01–0.05 = small effect, 0.06–0.13 = medium effect, ≥0.14 = large effect.

#### 3.2.1 Baseline

For sleep behaviour, significantly lower sleep quality was confirmed for shift workers compared to day workers (*η*^2^_p_ = 0.58—large effect). Thirty-one percent of shift workers and 13% of day workers fell into the *bad sleep* category (*χ*^2^(1) = 3.12, *p* = 0.077, *d* = 0.42—small effect). In the morning, shift workers went to bed at 21:48 h on average (SD = 3 h 6 min), day workers at 21:36 h (SD = 3 h 54 min, *t*(74) = 0.28, *p* = 0.782). Shift workers reported an average rising time of 04:25 h (SD = 30 min), day workers 05:48 h (SD = 54 min, *t*(74) = 7.90, *p* < 0.001, *d* = 2.05—large effect). At an average of 6 h 6 min (SD = 1 h), the sleep duration of shift workers was significantly shorter than that of day workers at 7 h 18 min (SD = 48 min, *t*(74) = 5.28, *p* < 0.001, *d* = 1.38—large effect). However, a distinction must be made between morning and night shift work: after the night shift, workers went to bed at 07:12 h on average (SD = 36 min) and rose at 12:54 h (SD = 1 h 12 min); they slept on average only 5 h 36 min (SD = 1 h 24 min) and thus 30 min less than during the morning shift (*t*(44) = 1.92, *p* = 0.061, *d* = 0.28). For shift workers, there was only a medium (*R* = 0.63) correlation for the PSQI score between morning and night shift.

#### 3.2.2 Follow-up

For the follow-up measurement, shift workers also reported lower sleep quality for the morning shift (*η*^2^_p_ = 0.11—medium effect) than day workers, although this did not change significantly in either group over the four-year period (*p* = 0.137); there was no significant interaction between the survey time points T1 and T5 (factor *time* * *group*, *p* = 0.155). However, there was a significant interaction effect for the sleep quality of shift workers after morning and night shifts at both survey times T1 and T5 (*η*^2^_p_ = 0.13—medium effect), according to which the PSQI score had practically deteriorated significantly after the night shift in 4 years (*d* = 0.46—small effect) and had reached the borderline of poor sleep quality (≥5 pts).

Among the shift workers, 27% reported poor sleep quality and 7% a chronic sleep disorder after the morning shift (T1: 31% poor sleep, *χ*^2^(1) = 8.99, *p* = 0.011, *d* = 0.74—medium effect); 31% of them reported poor sleep quality and 4% a chronic sleep disorder after the night shift (T1: 27% poor sleep, *χ*^2^(1) = 8.83, *p* = 0.007, *d* = 0.73—medium effect).

There were no significant changes between the baseline and follow-up measurement for bedtime (*F*(71) = 0.35, *p* = 0.563) and wake-up time (*F*(71) = 0.39, *p* = 0.534) in the morning shift resp. day work for any group. There was also no change in the duration of sleep for day workers, while for shift workers it was 18 min shorter on average for the follow-up. This effect was practically significant (*F*(42) = 7.90, *p* = 0.007, *η*^2^_p_ = 0.15—large effect) and was mainly observed on the night shift (*M* = 5 h 18 min, SD = 1 h 12 min).

The PSQI score correlated slightly (*R* = 0.49) between baseline and follow-up for shift workers on the morning shift and moderately (*R* = 0.60) for day workers, while a high correlation (*R* = 0.72) was observed between morning and night shift. *Age* did not have a significant influence on sleep quality in any group at any measurement time (morning shift vs. day work: *p* = 0.129, T1: SW: *R* = 0.08, DW: −0.02, T5: SW: *R* = 0.08, DW: 0.25).

### 3.3 State of health

**Figure 2:**
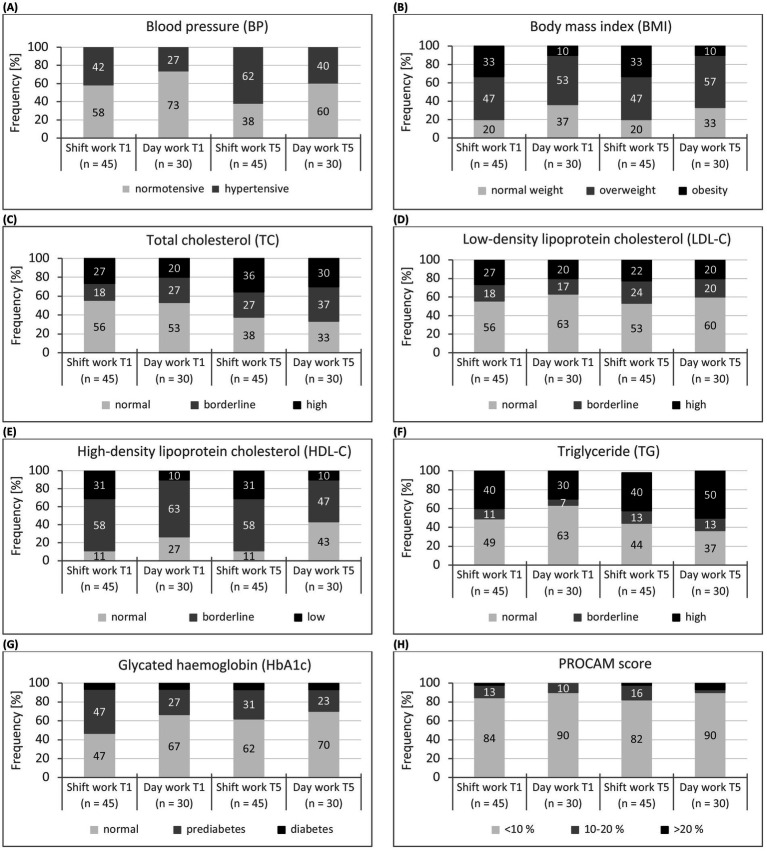
**(A-H)** Classification of cardiovascular health indicators (BP, BMI, TC, LDL-C, HDL-C, TG, HbA1c, PROCAM score) of shift and day workers at baseline and follow-up (T1, T5). *n*: sample size. Blood pressure: hypertensive: ≥140/90 mmHg (or/and) and intake of antihypertensive medication, normotensive: <140/90 mmHg (62).

The results for the assessment of cardiovascular health are shown in Table 4 and Figure 2.

**Table 4:** Main and interaction effects of cardiovascular health indicators of shift and day workers at baseline and follow-up.

Indicator		Shift work (*n* = 45)	Day work (*n* = 30)	Within- and between-subject effects		
				Test-value	*p*-value	Effect size
Systolic blood pressure [mmHg]	Baseline (*M* ± SD)	138.4 ± 13.4	129.8 ± 15.2	*t* = 2.55	0.013*	*d* = 0.60
	Follow-up (*M* ± SD)	133.2 ± 18.2	130.5 ± 14.8	*t* = 0.68	0.497	*d* = 0.16
		Group		*F* = 3.11	0.082	*η*^2^_p_ = 0.04
		Time		*F* = 0.12	0.733	*η*^2^_p_ < 0.01
		Time * group		*F* = 2.01	0.161	*η*^2^_p_ = 0.03
	Covariate	Age		*F* = 16.32	<0.001***	*η*^2^_p_ = 0.19
Diastolic blood pressure [mmHg]	Baseline (*M* ± SD)	82.2 ± 4.9	79.2 ± 7.8	*t* = 2.08	0.041*	*d* = 0.49
	Follow-up (*M* ± SD)	80.7 ± 7.7	80.5 ± 5.8	*t* = 0.10	0.920	*d* = 0.02
		Group		*F* = 1.11	0.296	*η*^2^_p_ = 0.02
		Time		*F* = 0.63	0.430	*η*^2^_p_ = 0.01
		Time * group		*F* = 2.77	0.101	*η*^2^_p_ = 0.04
	Covariate	Age		*F* = 16.05	<0.001***	*η*^2^_p_ = 0.18
Body mass index [kg/m^2^]	Baseline (*M* ± SD)	28.6 ± 4.4	25.7 ± 4.1	*t* = 2.88	0.003**	*d* = 0.68
	Follow-up (*M* ± SD)	28.9 ± 4.7	26.0 ± 3.7	*t* = 2.89	0.003**	*d* = 0.68
		Group		*F* = 7.74	0.007**	*η*^2^_p_ = 0.10
		Time		*F* = 1.02	0.315	*η*^2^_p_ = 0.01
		Time * group		*F* = 0.13	0.715	*η*^2^_p_ < 0.01
	Covariate	Age		*F* = 4.84	0.031*	*η*^2^_p_ = 0.06
Cholesterol [mmol/l]	Baseline (*M* ± SD)	5.3 ± 1.1	5.4 ± 1.1	*t* = 0.52	0.607	*d* = 0.12
	Follow-up (*M* ± SD)	5.4 ± 1.5	5.6 ± 1.2	*t* = 0.46	0.644	*d* = 0.11
		Group		*F* = 0.68	0.411	*η*^2^_p_ = 0.01
		Time		*F* = 1.35	0.492	*η*^2^_p_ = 0.02
		Time * group		*F* = 0.05	0.822	*η*^2^_p_ < 0.01
	Covariate	Age		*F* = 2.52	0.117	*η*^2^_p_ = 0.03
LDL-Cholesterol [mmol/l]	Baseline (*M* ± SD)	3.4 ± 1.0	3.3 ± 0.9	*t* = 0.75	0.455	*d* = 0.18
	Follow-up (*M* ± SD)	3.5 ± 1.0	3.2 ± 0.9	*t* = 1.46	0.148	*d* = 0.35
		Group		*F* = 0.97	0.329	*η*^2^_p_ = 0.01
		Time		*F* = 2.02	0.160	*η*^2^_p_ = 0.03
		Time * group		*F* = 1.07	0.305	*η*^2^_p_ = 0.02
	Covariate	Age		*F* = 1.20	0.276	*η*^2^_p_ = 0.02
HDL-cholesterol [mmol/l]	Baseline (*M* ± SD)	1.2 ± 0.3	1.4 ± 0.3	*t* = 2.54	0.013*	*d* = 0.60
	Follow-up (*M* ± SD)	1.2 ± 0.3	1.5 ± 0.4	*t* = 3.49	<0.001***	*d* = 0.82
		Group		*F* = 11.24	0.001***	*η*^2^_p_ = 0.14
		Time		*F* = 0.01	0.998	*η*^2^_p_ < 0.01
		Time * group		*F* = 1.37	0.246	*η*^2^_p_ = 0.02
	Covariate	Age		*F* = 0.07	0.799	*η*^2^_p_ < 0.01
Triglycerides [mmol/l]	Baseline (*M* ± SD)	2.2 ± 1.0	1.9 ± 0.9	*t* = 1.07	0.287	*d* = 0.25
	Follow-up (*M* ± SD)	2.3 ± 1.3	2.1 ± 0.8	*t* = 1.04	0.259	*d* = 0.25
		Group		*F* = 2.21	0.141	*η*^2^_p_ = 0.03
		Time		*F* = 2.91	0.092	*η*^2^_p_ = 0.04
		Time * group		*F* = 0.15	0.699	*η*^2^_p_ < 0.01
	Covariate	Age		*F* = 0.63	0.431	*η*^2^_p_ = 0.01
Glycated haemoglobin [mmol/mol]	Baseline (*M* ± SD)	39.5 ± 9.9	38.6 ± 8.1	*t* = 0.44	0.663	*d* = 0.10
	Follow-up (*M* ± SD)	38.1 ± 9.5	37.8 ± 7.3	*t* = 0.18	0.849	*d* = 0.04
		Group		*F* = 1.26	0.265	*η*^2^_p_ = 0.02
		Time		*F* = 6.59	0.012*	*η*^2^_p_ = 0.09
		Time * group		*F* = 1.87	0.176	*η*^2^_p_ = 0.03
	Covariate	Age		*F* = 16.68	0.001***	*η*^2^_p_ = 0.19
PROCAM score [pts]	Baseline (*M* ± SD)	30.3 ± 12.7	25.4 ± 14.4	*t* = 1.56	0.122	*d* = 0.37
	Follow-up (*M* ± SD)	33.8 ± 12.7	28.0 ± 16.6	*t* = 1.72	0.090	*d* = 0.40
		Group		*F* = 6.36	0.014*	*η*^2^_p_ = 0.08
		Time		*F* = 0.78	0.380	*η*^2^_p_ = 0.01
		Time * group		*F* = 0.57	0.451	*η*^2^_p_ = 0.01

*M* ± SD: mean ± standard deviation, *n*: sample size. *t*-test (test size: *t*-value, effect size: *d*). *rANCOVA* (test size: *F*-value, effect size: *η*^2^_p_): design: constant term + shift group + age (exception PROCAM score); within-subjects effect: time (T1, T5). *p*-value: significance (two-sided): *p < 0.05, **p < 0.01, ***p < 0.001. Effect size according to Cohen (69): *d*: <0.20 = no effect, 0.20–0.49 = small effect, 0.50–0.79 = medium effect, ≥0.80 = large effect. *η*^2^_p_: <0.01 = no effect, 0.01–0.05 = small effect, 0.06–0.14 = medium effect.

#### 3.3.1 Baseline

At baseline, blood pressure (*M* = 138/82 vs. 130/79 mmHg, *d* = 0.60—medium effect) and body mass index (*M* = 29 vs. 26 kg/m^2^, *d* = 0.68—medium effect) were significantly higher on average for shift workers than for day workers and significantly lower for HDL cholesterol (*M* = 1.2 vs. 1.4, *d* = 0.60—medium effect). The average blood pressure was classified as normal in both groups (62). However, one third (33%) of shift workers, but only 10% of day workers, took antihypertensives. The proportion of hypertensive individuals was only slightly higher among shift workers than among day workers (42% vs. 27%, *p* = 0.169). In MPOs who did not take antihypertensive drugs (SW: *n* = 30, DW: *n* = 27), the average blood pressure did not differ significantly between the two groups (*M* = 132/80 vs. 129/80 mmHg, *p* = 0.392/0.800). Similarly, there were no group differences between MPOs taking antihypertensive drugs (*M* = 150/87 vs. 133/75 mmHg, *p* = 0.065/0.264). The mean values of the body mass index in both groups had reached the overweight range (64). This affected about half of the shift workers (47%) and day workers (53%); obesity was present in 33% of the shift workers and 10% of the day workers (*d* = 0.64—medium effect). The mean values for HDL cholesterol in both groups had also reached borderline range (HDL-C: 1.5–1.0 mmol/l) (65). Only 11% of shift workers and around a quarter (27%) of day workers had acceptable HDL cholesterol levels.

The mean values for total cholesterol, LDL cholesterol, triglycerides and HbA1c did not differ significantly between the two groups (*p* > 0.05). However, the mean values for lipid status (TC, LDL-C, TG) had reached the borderline range in both groups (65). In each case, more than 40% of MPOs had elevated or high values for total cholesterol (≥5.2 mmol/l: 45%), LDL cholesterol (≥3.3 mmol/l: 41%) and triglycerides (1.7–2.2 mmol/l: 45%). The mean HbA1c values in both groups had also reached the borderline of prediabetes (64): 39–48 mmol/mol; 39% of MPOs were within the range of prediabetes and known diabetes was confirmed for 7%.

For the PROCAM score, there was a trend towards a higher cardiovascular risk for shift workers (*p* = 0.122). The high standard deviations (6%) indicated a very different risk. However, the mean values of the PROCAM score in both groups were in the low to moderate range (*M* = 30 vs. 25 pts), which predicts a low risk (0–47 pts: <10%) of a heart attack in the next 10 years (66). This was true for 87% of MPOs, 12% of MPOs were found to be at intermediate (48–56 pts: 10–20%) and 2% at high (>56 pts: >20%) cardiovascular risk (*p* = 0.638). On average, the PROCAM risk was 4.9% for shift workers and 4.0% for day workers (*t*(73) = 0.66, *p* = 0.509).

#### 3.3.2 Follow-up

After 4 years, no significant interaction effect for time * group was found for any of the health indicators analysed (*p* > 0.05). This suggests that these health indicators did not develop significantly differently in the two groups over the four-year period. The characteristics of the lipid metabolism and the risk of diabetes of the baseline measurement remained unchanged. However, a main effect for the *group* factor was found for the body mass index, HDL cholesterol and the PROCAM score. For these indicators, the significantly less favourable mean values of the shift workers at the baseline measurement were also found in the follow-up (T5: BMI: *M* = 29 vs. 26 kg/m^2^, *η*^2^_p_ = 0.10—medium effect, HDL-C: *M* = 1.4 vs. 1.5 mmol/l, *η*^2^_p_ = 0.14—large effect, PROCAM score: *M* = 34 vs. 28 pts, *η*^2^_p_ = 0.08—medium effect). For the HbA1c, there was a significant main effect of the *time* factor (*η*^2^_p_ = 0.08—medium effect), which showed a significant improvement in the average HbA1c values for the follow-up measurement.

Shift workers were also characterised by significantly lower mean blood pressure values for the follow-up. However, 42% of the shift workers were now taking antihypertensives (DW: 13%), which moderated blood pressure regulation. More than half (53%) of the MPOs were classified as persons with hypertension (T1:T5 (CC): SW: 62%, *p* < 0.001, DW: 40%, *p* = 0.018).

MPOs who did not take antihypertensive drugs at either measurement point (SW: *n* = 26, DW: *n* = 26) showed no significant difference in average blood pressure between the two groups at the follow-up measurement (*M* = 129/80 vs. 130/81 mmHg, *t*(50) = 0.856/0.827, *p* = 0.856/0.827). There were no significant differences for the factors *time* (*p* = 0.121/0.385), *group* (*p* = 0.468/0.254) or time * group (*p* = 0.447/0.382). Accordingly, blood pressure did not change significantly in these two groups over 4 years. Shift and day workers who were taking antihypertensive drugs at both measurement times had lower blood pressure at the follow-up measurement (*M* = 143/83 vs. 130/78 mmHg), but there were also no significant effects for the factors *time* (*p* = 0.526/0.474), *group* (*p* = 0.087/0.0.77) or time * group (*p* = 0.790/0.191).

As there were no effects of the *time* factor for any other health indicators, a comparable state can be assumed between baseline and follow-up. With the exception of blood pressure (SBP: *R* = 0.37, DBP: *r* = 0.29), this finding (*R* = 0.83–0.93) was confirmed in the correlation analysis.

*Age* had a significant effect on blood pressure, body mass index and HbA1c (*η*^2^_p_: SBP = 0.19—large effect, DBP = 0.18—large effect, BMI = 0.06—medium effect, HbA1c = 0.19—large effect). In both groups, the mean values of these indicators increased with *age* (T1: *R* = 0.22–0.61, T5: *R* = 0.21–0.67). *Age* had no influence on blood lipids (TC, LDL-C, HDL-C, TG: *p* > 0.05, T1: R = 0.01–0.18, T5: *R* = 0.02–0.09). The PROCAM score includes *age* itself as a risk factor.

### 3.4 Work-life balance

**Figure 3:**
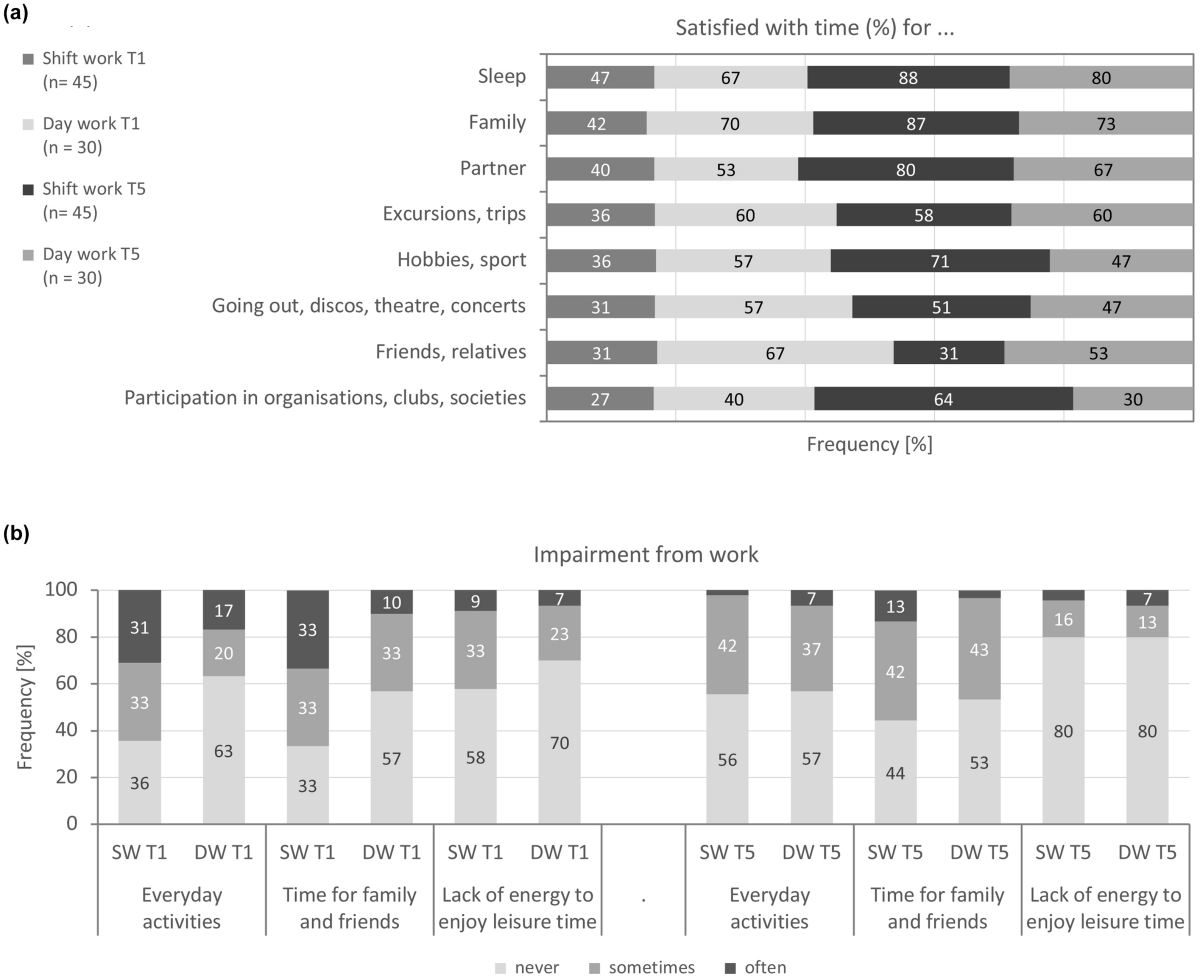
**(a)**Satisfactio with the time available for social and leisure activities of shift and day workers at baseline and follow-upT1, T5). **(b)** Impairment from work of shift and day workers at baseline and follow-up (T1, T5). SW: shift work, DW: day work.

Work-life balance was analysed using the two scales satisfaction and impairment of the questionnaire for shift workers (52) controlling for covariate (*children/household [number]*). The results of the work-life balance for the baseline and follow-up can be found in Table 5 and Figures 3a,b, whereby Figure 3a only reflects the work-related impairments to private life.

**Table 5:** Main and interaction effects of work-life-balance (scales satisfaction and impairment) of shift and day workers at baseline and follow-up.

Indicator		Shift work (*n* = 45)	Day work (*n* = 30)	Within- and between-subject effect		
				Test-value	*p*-value	Effect size
Satisfaction score [pts]	Baseline (M ± SD)	14.1 ± 5.4	10.9 ± 4.7	*t* = 2.67	0.009**	*d* = 0.63
	Follow-up (M ± SD)	10.0 ± 2.3	11.2 ± 3.6	*t* = 1.58	0.121	*d* = 0.41
		Group		*F* = 1.13	0.292	*η*^2^_p_ = 0.02
		Time		*F* = 11.30	0.001***	*η*^2^_p_ = 0.14
		Time * group		*F* = 19.37	<0.001***	*η*^2^_p_ = 0.21
	Covariate	Children/household [number]		*F* = 0.16	0.693	*η*^2^_p_ < 0.01
Impairment score [pts]	Baseline (*M* ± SD)	19.0 ± 3.0	19.9 ± 2.7	*t* = 1.38	0.172	*d* = 0.33
	Follow-up (*M* ± SD)	20.8 ± 2.4	21.4 ± 2.8	*t* = 1.05	0.299	*d* = 0.25
		Group		*F* = 0.61	0.436	*η*^2^_p_ = 0.01
		Time		*F* = 22.17	<0.001***	*η*^2^_p_ = 0.24
		Time * group		*F* = 0.41	0.526	*η*^2^_p_ = 0.01
	Covariate	Children/household [number]		*F* = 5.28	0.024*	*η*^2^_p_ = 0.07

*n*: sample size, *M* ± SD: mean ± standard deviation. *t*-test: (test size: *t*-value, effect size: *d*). *rANCOVA* (test size: *F*-value, effect size: *η*^2^_p_): design: constant term + group + children/household [number]; within-subjects effect: time (T1, T5). *p*-value: significance (two-sided): *p < 0.05, **p < 0.01, ***p < 0.001. Effect size according to Cohen (69): *d*: <0.20 = no effect, 0.20–0.49 = small effect, 0.50–0.79 = medium effect, ≥0.80 = large effect. *η*^2^_p:_ <0.01 = no effect, 0.01–0.05 = small effect, 0.06–0.14 = medium effect, ≥0.14 = large effect.

#### 3.4.1 Baseline

For the satisfaction score, a significant difference between shift and day workers was detectable at the baseline measurement (*d* = 0.63—medium effect), according to which shift workers were more dissatisfied with their available free time than day workers (*M* = 14 vs. 11 of 24 pts). This effect was consequently also reflected in the individual items of the satisfaction in social and leisure-related activities items (Figure 3a): about half of the MPOs reported being satisfied with the time available for sleep (*p* = 0.206). Shift workers complained most that they lacked time to participate in organisations or clubs; only about a quarter of them (DW: 40%) had regular time for such leisure activities (*p* = 0.120). Similarly, only around a third of shift workers were satisfied with the amount of time available for friends and relatives (DW: 67%, *d* = 0.76—medium effect), cultural events (DW: 57%, *d* = 0.76—medium effect), hobbies and sporting activities (DW: 57%, *d* = 0.69—medium effect), as well as excursions and travelling (DW: 60%, *p* = 0.085). About half were satisfied with the time they could spend with their partner (*p* = 0.195) and family (*p* = 0.106).

For the impairment score, there were no group differences at baseline (*p* = 0.172), i.e., at the start of the study, shift and day workers were impaired by their work to the same extent (*M* = 19 vs. 20 of 25 pts). This effect was only partially confirmed in the individual items (Figure 3b): for about half of MPOs, their private life was not affected by their work, for around a quarter *frequently* and *occasionally*, respectively (*p* = 0.061). A third of shift workers were also frequently and more than half occasionally unable to spend their time with family or friends as they wished (DW: 10% vs. 80%, *d* = 0.61—medium effect). 8% of all MPOs *frequently* and 29% *occasionally* lacked the energy to enjoy their free time; 63% did not experience this problem (*p* = 0.562).

#### 3.4.2 Follow-up

The time between the two survey points T1 and T5 did make a significant contribution to the explanation of the satisfaction with the time available for social and leisure activities scale for either shift or day workers (*η*^2^_p_ = 0.14—medium effect). There was no significant main effect for *group* (*p* = 0.292). Only the interaction of *time* * *group* had a practically significant effect on satisfaction (*η^2^*_p_ = 0.21—large effect), according to which satisfaction developed differently in the two groups over the 4 years. While shift workers were significantly more satisfied with their available time at the follow-up (*t*(44) = 6.36, *p* < 0.001, *d* = 0.94—large effect), it did not change for day workers compared to the baseline measurement (*t*(29) = 0.42, *p =* 0.675). In trend, shift workers were even more satisfied than day workers. The satisfaction score correlated only slightly (*R* = 0.49) between baseline and follow-up. There was no significant effect on satisfaction for the covariate *children/household [number]* (*p* = 0.693). For the follow-up measurement, there was only low correlations with the satisfaction score for this covariate (*children/household [number]: R* = 0.21).

The satisfaction effect for shift workers was confirmed in the individual items: more shift than day workers stated that they were satisfied with the time available for sleep (SW: 88%, DW: 80%, *d* = 0.64—medium effect) as well as participation in organisations or club life (SW: 64%, DW: 30%, *d* = 0.73—medium effect). Satisfaction was comparable in both groups with regard to the time available for the family (81%, *p* = 0.126) or partner (75%, *p* = 0.520). Satisfaction with the time available for cultural events (49%, *p* = 0.468), for hobbies and sporting activities (61%, *p* = 0.062), as well as excursions and travel (59%, *p* = 0.437) also did not differ between shift and day workers. However, only 31% of shift workers were still satisfied with their time for friends and relatives (DW: 53%, *p* = 0.719).

For the impairment score, there was neither a significant main effect for *group* (*p* = 0.436) nor an interaction effect for time * group (*p* = 0.526) but for the factor *time* (*η*^2^_p_ = 0.24—large effect). Accordingly, comparable changes can be assumed in both groups over the period of 4 years (*t*(74) = 1.05, *p* = 0.299). The changes at the follow-up indicated significantly less impairment in both shift and day workers (*M* = 21 of 25 pts, SW: *t*(44) = 4.46, *d* = 0.68—medium effect, DW: *t*(29) = 3.19, *d* = 0.58—medium effect). A medium correlation (*R* = 0.52) was found between baseline and follow-up for the impairment score. The *number of children to be cared in the household* had an influence on the impairments (*η*^2^_p_ = 0.07—medium effect). MPOs without children reported work-related or private impairments more frequently than those with at least one child. There was a low negative correlation with the covariate *children/household [number]* (*R* = −0.30). The individual items confirmed the clear decrease in work-related impairments in both groups.

## 4 Discussion

From an occupational health perspective, shift workers should be productive, resilient and satisfied with their work for as long as possible. Based on previous research findings, the study hypothesised at the beginning that MPOs working 12-h shifts over a period of 4 years would experience negative effects on their sleep quality and quantity (72, 73), cardiovascular health (74) and work-life balance (7). MPOs who worked daytime jobs served as a comparison group.

However, shift workers differed from day workers at the baseline measurement in terms of significantly poorer sleep quality, shorter sleep duration, less favourable values for blood pressure, body mass index, HDL cholesterol, PROCAM score and greater dissatisfaction with the time available for social, cultural and sporting activities.

In fact, the cardiovascular health status had not changed significantly in either group at the follow-up. For shift workers, the quality and quantity of sleep only decreased significantly after the night shift over a period of 4 years. Nevertheless, most shift workers (T5: 89%) were satisfied with the time they had available for sleep. For day workers, the comparatively higher sleep quality and longer sleep duration from the baseline measurement were also confirmed in the follow-up measurement. The effect on satisfaction was unexpected. Shift workers were significantly more satisfied with their available free time in the follow-up. This satisfaction was now even higher than that of day workers, for whom satisfaction was comparable at both times (T1, T5). In addition, both groups reported significantly fewer impairment*s* after 4 years, whereby this effect was moderated by the *number of children to be cared for in the household*. *Age* did not have a significant effect on sleep quality and quantity at any measurement *time* or in any *group*. However, with the exception of the lipid metabolism indicators, the known age effects were confirmed for the cardiovascular health indicators (BP, BMI, HbA1c, PROCAM score).

Shift workers slept on average 72 min less than day workers at baseline, whereby their average sleep duration after the night shift was still 30 min shorter than during the morning shift. For the follow-up, shift workers slept an average of only 5 h 18 min after the night shift, i.e., definitely too little. Daytime sleep after a night shift is also considered less restorative as it is more prone to disruption and rarely reaches the depth of night-time sleep (8, 43). According to Hulsegge et al. (17), around a third (35%) of shift workers and a quarter (27%) of day workers sleep less than seven hours. A representative survey of working people in Germany (*n* = 4,511, 31–60 years) also found that 12% of shift workers can be assumed to have insufficient rest (75). Research has shown that a lack of rest can lead to sleep disorders in the long term (75). Health experts therefore recommend that shift workers who sleep less than 7 h a day should take a power nap in between (13).

Although the additional days off outside the 12-h shifts offer employees opportunities to compensate for sleep deficits and lack of rest, they are probably not enough to fully compensate for them. In the study by Cunha et al. (8), only just under half (49%) of shift workers could imagine working in a 12-h shift system until retirement age. In addition, shift work appears to lead to a decrease in sleep quality after a short period of time (73).

Sleep disorders are also widespread in the general population. In Germany, 29% of all men (18–80 years) (57), and in an Austrian study 27% of all men (16- ≥ 70 years) have reported poor sleep quality (PSQI score >5 pts) (76). Chronic sleep disorders (PSQI score >10 pts) were not reported by Zeitlhofer et al. (76) and by Hinz et al. (57) for 5% of men.

In the study by Hinz et al. (57), the mean PSQI score for men in the general German population (18–59 years) was 4.3 pts In contrast to our sample, there was an age effect, according to which the PSQI score increased slightly with increasing age (<40 years: M = 3.9 pts, 50–59 years: *M* = 4.5 pts). In the study by Zeitlhofer et al. (76), the PSQI score also increased with increasing age in men from 2.5 (19–24 years) to 5.5 pts (55–59 years). Compared to these two studies, the day workers in the present study showed significantly better sleep quality with an average of 3.5 pts at baseline and 3.1 pts at follow-up. In contrast, shift workers reported slightly worse PSQI scores at the follow-up (T5: *M* = 4.8–5.1 pts) than in the general German population (57).

The correlations to the PSQI score between baseline and follow-up (SW: *R* = 0.49, DW: *R* = 0.60) and between morning and night shift (T1: *R* = 0.63, T5: *R* = 0.72) correspond to a variance explanation of 24–52%. Accordingly, only those who reported good sleep quality at baseline tended to also report this at follow-up. This also applies to the correlation between morning and night shift. Obviously, sleep behaviour is based on ‘third variables’ that occur independently of working hours. Sleep is a multidimensional construct and the causes of sleep problems are manifold.

With regard to cardiovascular health, the 12-h shift system had hardly any effect over a period of 4 years in this study. On the contrary, significant age effects were found. It is assumed that shift workers develop strategies to maintain their health and well-being that prepare the body for 12-h shifts or compensate for the effects of working past 12 h (8). To categorise the health status of shift and day workers, comparative studies that represent the general German male population and approximately the age range of the study have been used; they are as follows: German Health Interview and Examination Survey for Adults (DEGS1 study, 18–59 or 18-64 years) (77–80), PROCAM study (*n* = 5,389 men, average age: 47 years) (66) and Diabetes Cardiovascular Risk-Evaluation: Targets and Essential Data for Commitment of Treatment (DETECT study, *n* = 3,672 men, 40–65 years, average age: 53 years) (81).

From a preventive point of view, there is a need for action not only for shift workers but also for day workers with regard to cardiovascular risk. For example, almost two thirds (62%) of shift workers and 40% of day workers were considered to be hypertensive at the time of the follow-up, of whom only 43 and 13%, respectively, were taking antihypertensive drugs. The decrease in average blood pressure observed over the four-year period among shift workers is probably a medication effect. In future, occupational health programmes need to focus on increasing the proportion of people with hypertension treated with medication, regardless of whether they work only during the day or in shifts.

Furthermore, about half of the shift workers (47%) and day workers (57%) were found to be overweight and 33 and 10%, respectively, were obese. In addition, lipid metabolism disorders were present in both groups. Although the risk of a heart attack in the next 10 years could be classified as *low* for the majority of MPOs (T1:T5: <10%: 84 vs. 82%, DW: 90%), it had increased in both groups for the follow-up (*M* = 6.3 vs. 5.1 pts) although this was not practically significant. After 4 years, 4% of MPOs had a high PROCAM risk (T1: 1%).

In the study by Ohlander et al. (82), day workers had a lower prevalence of high blood pressure (8%) than shift workers (without night shift: 12%, night shift workers: 11%, rotating shift workers with nights: 10%). However, these prevalences are significantly lower than in our study and also lower than in the DEGS1 study, in which 15% of men had hypertension (77). The low prevalences are explained by the healthy worker effect and higher medical monitoring of employees compared to the general German population (82). Rashnuodi et al. (31), on the other hand, found elevated blood pressure in 44% of shift workers and 28% of day workers. Esquirol et al. (2) and Boini et al. (35) also postulate an increased risk of hypertension for shift workers, although the duration of exposure could influence this correlation. According to Su et al. (83), 12-h night work leads to an increase in blood pressure and heart rate as well as delayed blood pressure recovery. No association between shift work and high blood pressure was found in the longitudinal studies by Morikawa et al. (32), Hublin et al. (24) or Akbari et al. (28).

The average body mass index in this study is comparable to the mean values of Ohlander et al. (82) for both shift and day workers (SW with night work: *M* = 28 kg/m^2^, DW: *M* = 26 kg/m^2^). But the proportion of obese people among shift workers is significantly lower here (22–24%) than among our shift workers (33%), but slightly higher for day workers (12% vs. 10%). In contrast, only 20% of men in the DEGS1 study were affected by obesity (78). Results from reviews (34, 35, 84) and the longitudinal study by van Drongelen et al. (33) have confirmed that night work in particular is associated with an increased risk of being overweight and obesity. However, when the results were adjusted for potential confounders (e.g., age, physical activity), the evidence was insufficient (33). In the cohort studies by Dochi et al. (37) and Akbari et al. (28), the mean values for the body mass index were in the normal range (<25 kg/m^2^) and did not differ between shift and day workers. This result was also reported by Rashnuodi et al. (31), whereby the mean values of the body mass index (28 kg/m^2^) were in the overweight range. Consistent with these results, the prevalence of being overweight, but above all obesity, also increased with age among the men in the DEGS1 study (78).

In this study, the average increase in blood lipids occurred equally in both groups and could not be attributed to shift work, but rather to an age effect. There is consensus here with the results of Morikawa et al. (32), Dochi et al. (37), Akbari et al. (28) and Dong et al. (30), who also found no differences in lipid metabolism indicators between shift workers with night work and day work. In contrast, lipid metabolism disorders have been frequently found in reviews for shift and night workers (2, 28, 31, 37). Compared to the DEGS1 study (TC: *M* = 5.0 mmol/l, HDL-C: *M* = 1.3 mmol/l), the MPOs showed slightly higher mean values for total cholesterol (T1:T5: *M* = 5.3 vs. 5.5 mmol/l), for HDL cholesterol the mean values were comparable to those of the DEGS1 study (1.3 mmol/l) (80) and the PROCAM study (1.2 mmol/l) (66). Overall, 51% of those analysed in the DEGS1 study had borderline elevated levels of total cholesterol, 16% had high levels of total cholesterol and 21% had low levels of HDL cholesterol (80). In contrast, the sample shown here indicates a less favourable lipid metabolism status (T5: 31% elevated and 33% high TC values, 23% low HDL-C values). Boini et al. (35) found low HDL cholesterol in permanent night and rotating night shift workers, while Guo et al. (15) found no association between shift work and low HDL cholesterol. However, the link between lipid metabolism disorders and night shift work has not yet been clearly established (2, 25).

In terms of glucose metabolism, shift and day workers did not differ longitudinally; 12-h shift work did not have a negative effect on the HbA1c value over 4 years. On the contrary, the HbA1c value also increased with increasing age (T1-T5: *R* = 0.45–0.32). This finding contradicts the studies in which a higher prevalence of diabetes is postulated for shift and night workers compared to day workers (2, 38, 85). In contrast, results from the studies by Morikawa et al. (32), Akbari et al. (28) or Dong et al. (30), in which no differences were found between shift and day workers with regard to glucose metabolism, are consistent with the results given here. Unexpected was the decrease in the average HbA1c value for follow-up measurement in both groups. One possible explanation for this could be that the dietary habits of those affected had changed as a result of occupational health counselling.

Nevertheless, it is worth noting that 31% of shift workers and 23% of day workers had HbA1c values in the prediabetic range for the follow-up and 7% of all MPOs were affected by manifest diabetes. The prevalence of diabetes was therefore higher than in a comparable study with 12-h day and night shifts (85). Here, 5% of alternating shift workers (n = 4,150, average age: 45 years) and 3% of day workers (n = 5,976, average age: 44 years) reported diagnosed diabetes mellitus. After additional adjustment of the diabetes risk factor (Find-Risk-Score), the prevalence of diabetes was on average 23% higher for shift workers compared to day workers. However, this effect was not significant and was primarily attributed to the more pronounced Find-Risk risk profile of shift workers (85). The DEGS1 study found a diabetes prevalence of 3% for men (79). In both studies, the prevalence increased with increasing age (79, 85).

The PROCAM score used to summarise the assessment of cardiovascular health. The calculated risk of a cardiovascular event in the next 10 years differed significantly between the groups (*η*^2^_p_ = 0.08—medium effect) and increased with age. The age effect is also consistent with the findings from comparative studies (15, 66, 79). The average PROCAM score for the follow-up was 6.3% for shift workers and 5.1% for day workers, which is still in the low-moderate range (66). In the PROCAM study, an average PROCAM risk of 6.8% was determined (66). In comparison, a significantly higher mean value of 9.2% was determined for the PROCAM score in the DETECT study (81).

Our findings are consistent with those of the meta-analysis by Vyas et al. (19), according to which shift workers have a higher risk of cardiovascular disease than day workers. However, the moderate increase in PROCAM risk for the follow-up is interpreted more as an age effect and less as a shift system effect, i.e., the fully continuous 12-h shift system does not appear to have an effect on cardiovascular risk. In the longitudinal study by Guo et al. (15), no clear tendencies for an increased cardiovascular risk among shift workers were recognisable for men over a period of 22 years. Yong et al. (26) also found no evidence of an increased cardiovascular risk among 12-h shift workers in industrial production.

From a medical point of view, it is worrying that a significant proportion of the cardiovascular risks in the MPOs were only diagnosed as a result of the study. Most of them were unaware of their risk factors or diseases. As a result, they were not receiving medical treatment. This is where the potential lies for holistic occupational health care in Germany, which takes into account not only the working conditions but also the individual interactions between work and physical and mental health, which can jeopardise employability.

The predictability and plannability of working hours are the key to work-life balance. The 12-h shift system means that shift workers are exposed to work-related stress for longer, but have more continuous time for relaxation, family and non-work activities (8). In addition, there is the financial advantage of shift allowances, less travelling time and costs, and free time at unusual times of the day (8).

Satisfaction with the time available for social and leisure activities changed significantly differently in the groups between the two survey dates. While the shift workers were more dissatisfied at the baseline measurement, a positive satisfaction effect was registered over the four-year period. In addition, private life was hardly affected by shift work. Accordingly, the decision in favour of a 12-h shift schedule can be useful for shift workers at certain stages of life (e.g., with young children), while health aspects are of secondary importance in this context (8). On the other hand, shift workers felt the sleep-related effects and occupational wear and tear of a shift system with night work. Irrespective of this, the benefits of the additional days off of a 12-h shift system described by Berkam et al. (86) appear to contribute significantly to the satisfaction of shift workers. Overall, the findings point to a very diverse family and social private life for shift workers.

The family and social conditions of the shift and day workers were comparable at both measurement times: just under 80% of MPOs lived in a partnership. Around half of them were childless and only 8 and 10% of the shift workers had children up to the age of three in their household, so that hardly any adverse effects were expected.

In essence, the development of cardiovascular health over the course of a lifetime must be assumed to be multi-causal. The underlying mechanisms for the health consequences of shift work are not yet fully understood (2, 87). So far, no causal relationship between shift work, disturbed sleep and health risks has been proven (16, 25), even if numerous studies suggest this relationship. It is evident that general resilience decreases and illnesses increase with age.

The influence of shift work on cardiovascular and metabolic diseases is most frequently explained by desynchronized circadian rhythms, sleep disturbances, unhealthy diet, psychosocial work stress and social inequality (2, 25). Although the body can adapt to changes in the daily routine in the short term, recurring disruptions to natural biological rhythms such as night work can lead to sleep disturbances and have health implications (including weight gain, increased lipids, coronary heart disease, type II diabetes) (16, 19). The consumption of energy-dense foods during the night shift also appears to favour the development of dyslipidaemia and insulin resistance (88). Lifestyle changes include unhealthier eating habits (22, 25), smoking (22, 35), reduced social (43, 44) or physical activity (22, 36, 40) have been discussed as further possible causes for the development of cardiovascular and metabolic diseases. In this study, more than half (57%) of the shift workers were regularly active in sports (DW: 80%) and one third (33%) were smokers (DW: 30%). In previous research, only a few studies have focused on measures to improve the health problems of shift workers. The current review by Wasiewicz-Ciach et al. (13) summarises measures that shift workers can take to better protect their health.

In summary, the study showed that a 12-h rotating shift system is associated with poorer sleep quality and shorter sleep duration compared to working 8 h during the day. Although these effects worsened over the four-year observation period, this only occurred after night shifts. This means that the hypothetical negative impact of a 12-h shift system on sleep can only be partially confirmed. During the same period, shift workers’ satisfaction with the 12-h shift system increased, reaching a level comparable to that of day workers. In addition, both groups reported a decrease in work-related impairments. These results lead to the rejection of the hypotheses regarding the negative impact of a 12-h shift system on work-life balance. In contrast, the cardiovascular health of the MPOs in both groups did not change over the observation period. This hypothesis must therefore also be rejected.

### 4.1 Strengths

The originality of the study consists in the fact that data of a homogenous occupational group in a clearly defined shift system were examined in a longitudinal design over 4 years and possible covariates were checked. In addition, employees who work only during the day and whose occupations are largely comparable proved to be an important comparison group. Most of the studies on shift work have been based on a cross-sectional design with partially heterogeneous samples in which shift and night work are defined inconsistently and different shift systems are mixed. The studies have had different levels of evidence, in which covariables (e.g., nutrition, alcohol, smoking, exercise, sleep) have not been considered or in an undifferentiated way. Due to these limitations in the methodological quality of the studies, the conclusions drawn from them regarding the effects of shift work on sleep, health or work-life balance may not always have been justified and risks may have been over-interpreted.

In the present study, the shift work factor was added to the PSQI questionnaire and the retrospective period for recording sleep quality and quantity was again adapted to the original version – it refers to the last 4 weeks prior to the survey (52). The German version of the PSQI (56) was based on a period of only 2 weeks, which limits the comparability with international studies.

### 4.2 Limitations

It cannot be ruled out that the results are overlaid by different selection effects. For example, employees with good physical and mental adaptability (shift work tolerance) could remain in shift work with night work for longer than those who do not tolerate the stresses of shift work and night work or who have health problems (healthy worker effect, self-selection) (43). Paradoxically, such effects can make shift workers appear healthier than ‘normal day workers’ or the total population of the same age (89). Since participation in the occupational health check-up was voluntary, it is possible that primarily those who were health-conscious and healthy took advantage of the examinations, i.e., the health risks of this sample could still have been underestimated.

The calculation of the sample size was based on a large effect size, which was not confirmed by the present results. Therefore, the study’s power in detecting small differences in the outcomes between shift and day workers over time could be limited.

With regard to data collection, the results are subject to the well-known limitations of self-assessments using questionnaires (including distortions due to social desirability, response tendencies, memory deficits).

The body mass index is not accurate enough to determine individual health risks, as gender, age, origin, lifestyle, body composition, and fat and muscle mass are not included in its calculation. It can misestimate the health risk for overweight people and should therefore be supplemented by measures to estimate the fat distribution pattern in the body (waist-hip ratio or waist-to-height ratio). Regardless of this, the body mass index remains the decisive measure for diagnosing obesity in Germany (64).

In order to use the DEGS1 studies as comparative data for the general German population, the age ranges for the present study were adjusted and recalculated. Exact comparisons require cross-calibrations of the measurement results, which are not available for pre-analytical conditions even with comparable measurement methods.

The present study is based on only one sample of 75 MPOs. Therefore, the results can only be generalised to other occupational groups that work in 12-h shifts to a limited extent.

## 5 Conclusion

The consequences of shift work are complex and depend on the specifics of the shift models. For acceptance among employees, it is crucial that shift work and non-work needs can be satisfactorily reconciled. In this respect, 12-h rotating shift systems seem to offer advantages over regular eight-hour day work. However, the shorter sleep duration and reduced sleep quality after night work are additional limitations that endanger the long-term employability of shift workers, especially older workers. To counteract this, workers must be offered sufficiently long rest periods after night work to compensate for sleep deficits.

There is no ideal shift model that can eliminate all the unfavourable effects of night work on employees. Night work is always accompanied by a desynchronization of the circadian and social rhythms. It is therefore necessary to find solutions that promote the health of shift workers. The 12-h shift system and the associated distribution of days off and work days affect the quality and quantity of sleep.

Since sleep disorders can reduce performance and quality of life and lead to long-term health problems, they must be detected and treated at an early stage. In this context, occupational medicine can provide a valuable supplement to care provided by general practitioners and specialists. Its task is to limit adverse effects of night shift work on sleep, health and work-life balance. Individual measures for better sleep hygiene could be just as beneficial as an effective company health management system with a focus on sleep and cardiovascular health in achieving a successful outcome in prevention.

The findings on cardiovascular health indicate a need for prevention and medical care for both shift and day workers. Monitoring the prevalence of hypertension, overweight and obesity would be relevant to health, but should be placed in the context of a healthy lifestyle.

## Data Availability

The datasets presented in this article are not readily available because the dataset supporting the conclusions of this article is not included in the article on the basis of agreed data protection commitments to the participants. Requests to access the datasets should be directed to reingard.seibt@uni-rostock.de.

## Glossary

ArbZG: Working Hours Act
BafA: Federal Employment Agency
BGN: German Government Safety Organisation Foods and Restaurants
BMI: body mass index
BP: blood pressure
CI: confidence interval
DAG: German Obesity Society
DBP: diastolic blood pressure
DEGS1: German Health Interview and Examination Survey for Adults
DETECT: Diabetes Cardiovascular Risk-Evaluation: Targets and Essential Data for Commitment of Treatment
DW: day worker
HbA1c: glycated haemoglobin
HDL-C: high-density lipoprotein cholesterol
LDL-C: low-density lipoprotein cholesterol
MPO: machine and plant operators
OR: odds ratio
SBP: systolic blood pressure
PROCAM: Prospective Cardiovascular Münster
PSQI: Pittsburgh Sleep Quality Index
SPSS: Statistical Package for the Social Science
SW: shift worker
TC: total cholesterol
TG: triglycerides
